# An approximate Bayesian significance test for genomic evaluations

**DOI:** 10.1002/bimj.201700219

**Published:** 2018-08-12

**Authors:** Dörte Wittenburg, Volkmar Liebscher

**Affiliations:** ^1^ Institute of Genetics and Biometry Leibniz Institute for Farm Animal Biology Wilhelm‐Stahl‐Allee 2 D‐18196 Dummerstorf Germany; ^2^ Department of Mathematics and Computer Science University of Greifswald Walther‐Rathenau‐Str. 47 D‐17489 Greifswald Germany

**Keywords:** conditional expectation, dominance, epistasis, genetic architecture, SNP

## Abstract

Genomic information can be used to study the genetic architecture of some trait. Not only the size of the genetic effect captured by molecular markers and their position on the genome but also the mode of inheritance, which might be additive or dominant, and the presence of interactions are interesting parameters. When searching for interacting loci, estimating the effect size and determining the significant marker pairs increases the computational burden in terms of speed and memory allocation dramatically. This study revisits a rapid Bayesian approach (fastbayes). As a novel contribution, a measure of evidence is derived to select markers with effect significantly different from zero. It is based on the credibility of the highest posterior density interval next to zero in a marginalized manner. This methodology is applied to simulated data resembling a dairy cattle population in order to verify the sensitivity of testing for a given range of type‐I error levels. A real data application complements this study. Sensitivity and specificity of fastbayes were similar to a variational Bayesian method, and a further reduction of computing time could be achieved. More than 50% of the simulated causative variants were identified. The most complex model containing different kinds of genetic effects and their pairwise interactions yielded the best outcome over a range of type‐I error levels. The validation study showed that fastbayes is a dual‐purpose tool for genomic inferences – it is applicable to predict future outcome of not‐yet phenotyped individuals with high precision as well as to estimate and test single‐marker effects. Furthermore, it allows the estimation of billions of interaction effects.

## 1 INTRODUCTION

In animal breeding, molecular markers (e.g., single nucleotide polymorphisms; SNPs) are incorporated into statistical models to reach an improved genomic evaluation of animals. This leads to more precisely estimated breeding values of not‐yet phenotyped animals and, if selection of animals is based on genomic breeding values instead of traditionally estimated breeding values, breeding costs can be drastically reduced due to the shortened generation intervals (e.g. Schaeffer, 2006). Such genomic data are also extremely valuable for elucidating the genetic architecture of some trait. Not only the size of the genetic effect and the position on the genome, but also the mode of inheritance, which might be additive or dominant, and the presence of interactions are relevant for understanding the impact of a causative variant. Though there is little evidence that interactions (epistasis) contribute much to genetic variation in most populations (Hill, Goddard, & Visscher, 2008; Phillips, 2008), the revelation of epistasis may contribute to fill the gap of “missing heritability” (Zuk, Hechter, Sunyaev, & Lander, 2012).

In a typical situation of genomic evaluations, there are many more predictor variables (*p* SNPs) than observations (*n* animals); this makes testing of SNP effects a challenge. SNPs with significant impact on a trait can be identified with genome‐wide association studies (GWAS) typically based on successive single‐SNP investigations – one SNP is tested at a time while “walking” along the genome. Those SNPs being in high linkage disequilibrium (LD) with a causative variant will likely have a tiny *p*‐value implying a strong association with the trait. The high LD among SNPs complicates the correct identification of a causative variant, and this kind of testing also suffers from multiplicity. Several specific methods exist for controlling the rate of false‐positive detection. Ideally, the number of independent tests accounts for the LD between SNPs as was proposed by Gao, Starmer, and Martin (2008) in their simple M‐method. Alternatively, a score‐based test statistic accounting for the correlation between SNPs yields a ranked order of SNPs (Zuber, Duarte Silva, & Strimmer, 2012). Then, the top‐ranked SNPs can be taken for further genetic investigation.

Beside different strategies for suitable *p*‐value correction, using a statistical model that considers all SNPs jointly is a promising option. To study the genetic effect captured by SNPs, each with two alleles A and B, a whole‐genome regression model is set up (e.g. de los Campos, Hickey, Pong‐Wong, Daetwyler, & Calus, 2013). A trait is regressed onto the number of reference SNP alleles at all loci simultaneously. Such a model can be used for the estimation of SNP effects and/or for the selection of relevant SNPs throughout the genome. Excluding any nuisance effects the whole‐genome regression model can be written as

(1) y=Xg+e,

with $y={\left(y_{1},\ldots ,y_{n}\right)}^{\prime }$ being the vector of observable traits of *n* individuals centred by the population mean and $g={\left(g_{1},\ldots ,g_{p}\right)}^{\prime }$ the effect at *p* SNPs. The (n×p) design matrix $X=\{X_{i,j}\}$ contains the genotype codes at locus j∈{1,…,p} for individual i∈{1,…,n}, where 1 and −1 indicate homozygous genotypes AA and BB, respectively, and the heterozygote is coded as 0. Let A denote the minor allele with minor allele frequency (MAF) $f_{j}\leq 1/2$. The MAF is assumed to be known or may be estimated from SNP genotypes using, for example, the method of moments as $f_{j}=\frac{1}{2n}\sum_{i=1}^{n}\left(X_{i,j}+1\right)$. The residual errors in $e={\left(e_{1},\ldots ,e_{n}\right)}^{\prime }$ are assumed to be independent and normally distributed with zero mean and variance $\sigma_{e}^{2}$.

Selection‐and‐shrinkage approaches allow the simultaneous selection of those predictors that sufficiently map the trait and the estimation of their effect sizes. They are often implemented in a Bayesian (e.g. George & McCulloch, 1993; Habier, Fernando, Kizilkaya, & Garrick, 2011; Meuwissen, Hayes, & Goddard, 2001) or Expectation‐Maximization framework (Chen & Tempelman, 2015). Such a multiple‐locus model gains higher power than single‐SNP GWAS; it is therefore the preferred choice (Hoggart, Whittaker, De Iorio, & Balding, 2008). When based on a Markov‐chain‐Monte‐Carlo (MCMC) sampling scheme, Bayesian approaches, however, may become too time‐consuming and memory‐demanding if the model dimension is high (Meuwissen, Hayes, & Goddard, 2001; Pérez, de los Campos, Crossa, & Gianola, 2010). As an alternative to MCMC sampling, effect sizes can be approximated using a variational Bayes (vbay) approach, in which the posterior probabilities are approximated through factorisation. A vbay approach is competitive to MCMC‐based methods in terms of estimating the effect size of SNPs and detecting the significant loci but it requires only a fraction of computing time (Li & Sillanpää, 2012). Applied to real and simulated data, the vbay SNP‐selection approach of Logsdon, Hoffman, and Mezey (2010) could identify even SNPs with weak association to a trait. A rapid approximation of SNP‐effect estimates can also be derived by a Bayesian approach in which the conditional expectation of SNP effects is calculated iteratively (Meuwissen, Solberg, Shepherd, & Woolliams, 2009). Initially developed under pure additivity, this “fastbayes” has been extended to include dominance and epistatic effects (Wittenburg, Melzer, & Reinsch, 2011). That study showed a similar precision of genetic value prediction of fastbayes and an MCMC‐based version.

Model complexity and dimension are still an issue, also in times of high‐performance computing. Particularly, as whole‐genome sequence data are available, a causative variant shall be pinpointed to a specific base pair among millions of SNPs. Hence a fast algorithm is required to analyze the effects of all SNPs in a feasible time. The objective of the present study is to follow up the fastbayes approach and to incorporate a suitable measure of significance for testing the SNP effects. Both aspects of genomic inferences are evaluated – the ability to detect relevant loci and to predict genetic values – when different kinds of genetic effects are present (additive, dominance, epistasis). In Section 2, the components of the fastbayes approach are presented and a marginal test for genetic effects is developed. The extended approach is applied to simulated and real data, which are described in Section 3. Section 4 explains the implementation and involved software. The computing details are addressed in Section 5. Then, in Section 6, results of genomic evaluations for different parameter settings are presented. The performance of fastbayes is reviewed in Section 7, and further extensions are outlined.

## 2 METHODS

### 2.1 Summary of fastbayes

The following investigations are based on model (1) but the entries in *X* contain the standardised genotype codes. They are centred and scaled such that $X_{i,j}$ is $2\left(1-f_{j}\right)/s_{j}$ and $-2f_{j}/s_{j}$ for homozygous AA and BB, respectively, and $\left(1-2f_{j}\right)/s_{j}$ for heterozygous individuals with scaling term $s_{j}=\sqrt{2f_{j}\left(1-f_{j}\right)}$.

The approximate Bayesian method “fastbayes” is now further considered to include a testing procedure. According to Meuwissen et al. (2009), the idea of this iterative approach is based on a one‐locus model,

(2) $$\begin{array}{c} y=xg+e\;, \end{array}$$

where $x=X_{j}$, the *j*‐th column of *X*, and $g=g_{j}$ at a single locus j∈{1,…,p}.

The likelihood function p(y|g) is specified as a normal distribution with mean ***x*** *g* and covariance matrix $I\sigma_{e}^{2}$. The prior distribution of genetic effects is assumed to be a mixture of a Laplace distribution and a point mass at zero (δ_0_) with P(g=0)=1−γ. The formal prior density is

$$\begin{array}{ccc} p(g|\gamma ) & = & \frac{1}{2}\gamma \lambda \exp\left(-\lambda |g|\right)+\left(1-\gamma \right)\delta_{0}\;. \end{array}$$

The choice of the hyperparameters γ and λ will be discussed later.

A point estimate of the genetic effect is determined as the posterior expectation $\hat{g}=\text{E}\left(g|y\right)$, which is given in closed form (Meuwissen et al., 2009), that is

$$\begin{array}{ccc} & & \text{E}\left(g|y\right)=\frac{T_{1}\Theta_{U}\left(0;Y^{-},\sigma^{2}\right)+T_{2}\Theta_{L}\left(0;Y^{+},\sigma^{2}\right)}{T_{1}+T_{2}+T_{3}}\\ & & \begin{array}{cc} \text{with} & T_{1}=\exp\left(-\lambda Y\right)\left[1-\Phi \left(0;Y^{-},\sigma^{2}\right)\right],\\ & T_{2}=\exp\left(\lambda Y\right)\Phi \left(0;Y^{+},\sigma^{2}\right),\\ & T_{3}=\frac{2(1-\gamma )}{\gamma \lambda }\exp\left(-\frac{1}{2}\lambda^{2}\sigma^{2}\right)\phi \left(0;Y,\sigma^{2}\right), \end{array} \end{array}$$

where $\sigma^{2}={\left(x^{\prime }x\right)}^{-1}\sigma_{e}^{2}$, $Y={\left(x^{\prime }x\right)}^{-1}x^{\prime }y$ and $Y^{\pm }=Y\pm \lambda \sigma^{2}$. The $\Theta_{U}\left(0;\mu ,\sigma^{2}\right)$ and $\Theta_{L}\left(0;\mu ,\sigma^{2}\right)$ are the expected value of an upper and lower truncated normal distribution $N(\mu ,\sigma^{2})$, respectively, with truncation point zero. The $\Phi (x;\mu ,\sigma^{2})$ denotes the normal distribution function evaluated at some point *x*, and $\phi (x;\mu ,\sigma^{2})$ is the normal density function.

In order to analyze *p* loci simultaneously, the model (2) is fitted iteratively to a marginalized component, similar to Meuwissen et al. (2009) and Wittenburg et al. (2011). The trait ***y*** is corrected for all SNP effects except the one under investigation,

$$\begin{array}{ccc} y_{j} & = & y\;-\sum_{i=1,i\neq j}^{p}X_{i}g_{i}\;=\;X_{j}g_{j}+e\;\text{for}\;j=1,\ldots ,p\;. \end{array}$$

This is done using a Gauss‐Seidel‐like algorithm. Set $g_{j}^{\left(0\right)}=0$ ∀j. In iteration k=1,2,…,

(3) $$\begin{array}{c} \begin{array}{c} y_{j}^{\left(k\right)}=y-\sum_{i=1}^{j-1}X_{i}{\hat{g}}_{i}^{\left(k\right)}-\sum_{i=j+1}^{p}X_{i}{\hat{g}}_{i}^{\left(k-1\right)}\;\text{and}\\ {\hat{g}}_{j}^{\left(k\right)}=\text{E}\left(g_{j}|y_{j}^{\left(k\right)}\right) \end{array} \end{array}$$

are calculated for all j=1,…,p until relative changes are small,

$$\begin{array}{ccc} \frac{∥{\hat{g}}_{j}^{\left(k\right)}-{\hat{g}}_{j}^{\left(k-1\right)}∥}{∥{\hat{g}}_{j}^{\left(k\right)}∥} & < & 10^{-4}\;. \end{array}$$

Model (1) can be further extended to include dominance and two‐locus epistatic effects; details are presented in Wittenburg et al. (2011). Then the design matrix *X* consists of three groups of columns, one for each kind of effect, that is $X=(X_{a},X_{d},X_{e})$ comprising the standardized genotype codes for additive, dominance and epistatic effects. For dominance effects, the columns in $X_{d}$ are coded according to Falconer's model in a random mating population in Hardy–Weinberg equilibrium (Falconer & Mackay, 1996, p.118) and scaled afterwards. Then, at a specific locus *j*, $-2{\left(1-f_{j}\right)}^{2}/s_{j}^{2}$ is used for homozygous AA, $-2f_{j}^{2}/s_{j}^{2}$ for BB individuals and $2f_{j}\left(1-f_{j}\right)/s_{j}^{2}$ for heterozygous individuals. Four different types of epistatic effects can be distinguished: additive× additive, additive× dominance, dominance× additive, and dominance× dominance, leading to 2p(1−p) additional effects. A column in $X_{e}$ coding for an epistatic effect contains the product of codes for the corresponding main effects. For instance, an additive× dominance effect at locus pair *i* and *j* is coded as $-2\left(1-f_{i}\right)/s_{i}\cdot 2f_{j}^{2}/s_{j}^{2}$ if an individual is homozygous AA at locus *i* and homozygous BB at locus *j*. Again the Gauss‐Seidel‐like algorithm (3) is applied to ***y*** that is corrected for all other effects except the current one in order to estimate the different kinds of genetic effects.

In a real data analysis, also nongenetic factors affecting the trait have to be considered. Thus, model (1) is extended to account for potential fixed effects $b={\left(b_{1},\ldots ,b_{q}\right)}^{\prime }$ with (n×q) design matrix *W*,

$$\begin{array}{ccc} y & = & Wb+Xg+e\;. \end{array}$$

Like in Meuwissen et al. (2009) and Wittenburg et al. (2011), a type of empirical Bayes method is employed to estimate the fixed effects and $\sigma_{e}^{2}$ in iteration *k* of the Gauss‐Seidel‐like algorithm (3) as

$$\begin{array}{ccc} {\hat{b}}^{\left(k\right)} & = & {\left(W^{\prime }W\right)}^{-1}W^{\prime }y^{*}\;\text{with}\;y^{*}=y-X{\hat{g}}^{\left(k\right)}\;,\\ \sigma_{e}^{2(k)} & = & \frac{1}{n-q}e^{{\left(k\right)}^{\prime }}e^{\left(k\right)}\;\text{with}\;e^{\left(k\right)}=y-W{\hat{b}}^{\left(k\right)}-X{\hat{g}}^{\left(k\right)}\;. \end{array}$$

Then the estimation of genetic effects continues while ***y*** is corrected for the current estimates of fixed effects.

### 2.2 Marginal test for genetic effects

As a novel contribution, the significance of SNP‐effect estimates is investigated using a measure of evidence similar to De Braganca Pereira & Stern (1999). Starting with model (2), a fully Bayesian significance test is proposed to test the null hypothesis *H*_0_: g=0. For this purpose, the credibility κ of the highest posterior density (HPD) interval that is right to zero if $\hat{g}>0$ or left to zero if $\hat{g}<0$ is determined, that is

$$\begin{array}{ccc} \kappa =\text{P}(t_{1}<g<t_{2}|y) & = & \int_{\left(t_{1},t_{2}\right)}p\left(g|y\right)dg\;. \end{array}$$

In case of $\hat{g}>0$, the interval borders are $t_{1}=0$ and

$$\begin{array}{ccc} t_{2} & = & \max_{g>0}\left\{g:\;p\left(g|y\right)\geq p\left(0|y\right)\right\}, \end{array}$$

else if $\hat{g}<0$, $t_{2}=0$ and

$$\begin{array}{ccc} t_{1} & = & \min_{g<0}\left\{g:\;p\left(g|y\right)\geq p\left(0|y\right)\right\}\;. \end{array}$$

Then significance of *g* is inferred if 1−κ≤α, with an appropriately chosen type‐I error α. Let 1 denote the indicator function. The posterior probability is analytically derived as

$$\begin{array}{ccc} \text{P}(g<t|y) & = & \frac{1}{p(y)}\int_{\left(-\infty ,t\right)}p\left(y|g\right)p\left(g|\gamma \right)\;dg=\\ & = & \frac{1}{p(y)}\int_{\left(-\infty ,t\right)}\left[f_{1}\left(g\right)1_{\left\{g<0\right\}}+f_{2}\left(g\right)\delta_{0}\left(g\right)+f_{3}\left(g\right)1_{\left\{g>0\right\}}\right]dg, \end{array}$$

using the functions

$$\begin{array}{ccc} f_{1}\left(g\right) & = & \frac{1}{2}\gamma \lambda \exp\left(\frac{1}{2}\lambda^{2}\sigma^{2}+\lambda Y\right)\phi \left(g;Y^{+},\sigma^{2}\right),\\ f_{2}\left(g\right) & = & \left(1-\gamma \right)\phi \left(g;Y,\sigma^{2}\right),\\ f_{3}\left(g\right) & = & \frac{1}{2}\gamma \lambda \exp\left(\frac{1}{2}\lambda^{2}\sigma^{2}-\lambda Y\right)\phi \left(g;Y^{-},\sigma^{2}\right). \end{array}$$

Furthermore, the denominator is

$$\begin{array}{ccc} p(y) & = & \frac{1}{2}\gamma \lambda \exp\left(\frac{1}{2}\lambda^{2}\sigma^{2}\right)\left(T_{1}+T_{2}\right)+\left(1-\gamma \right)\phi \left(0;Y,\sigma^{2}\right)\;. \end{array}$$

**Figure 1:**
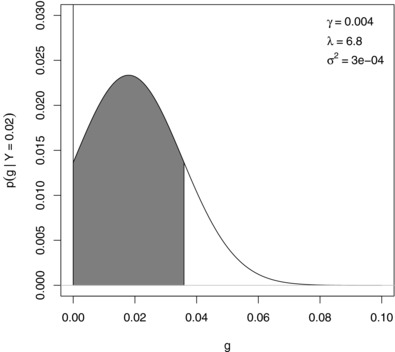
Example of a highest posterior density interval tangent to zero with arbitrary parameters mentioned within the graph

As the posterior density p(g|y) is bimodal due to the spike at zero, the posterior distribution function has a jump discontinuity at zero with step height $\frac{1}{p(y)}f_{2}\left(0\right)$; the smaller $Y={\left(x^{\prime }x\right)}^{-1}x^{\prime }y$, the larger the step height. For larger values of *Y* the density is approximately unimodal and symmetric (Meuwissen et al., 2009). This discontinuity, however, does not affect the calculation of κ because the region tangent to zero is of interest here. An example presenting the HPD interval is given in Figure 1.

In the *p*‐locus case and for the different kinds of effects, a marginal testing problem is considered for each SNP effect $g_{j}$. The corresponding credibility $\kappa_{j}$ is determined conditionally on ***y***_*j*_ when convergence of the iterative approach (3) has been reached.

For comparison, the Bayes factor is calculated as the ratio of marginal likelihoods of the full model over the reduced model, that is $g_{j}=0$, as

$$\begin{array}{ccc} B_{j} & = & \frac{\exp\left[-\frac{1}{2\sigma_{e}^{2}}{\left(y_{j}-X_{j}{\hat{g}}_{j}\right)}^{\prime }\left(y_{j}-X_{j}{\hat{g}}_{j}\right)\right]}{\exp\left(-\frac{1}{2\sigma_{e}^{2}}y_{j}^{\prime }y_{j}\right)}=\\ & = & \exp\left[-\frac{1}{2\sigma_{e}^{2}}\left(-2y_{j}^{\prime }X_{j}{\hat{g}}_{j}+{\hat{g}}_{j}X_{j}^{\prime }X_{j}{\hat{g}}_{j}\right)\right]. \end{array}$$

Following Jeffrey (1961), a substantial effect is reported if $B_{j}>3$.

## 3 DATA STRUCTURE AND EVALUATION CRITERIA

### 3.1 Simulation study

Genomic evaluations were implemented based on simulated data with realistic genetic structure, as described and used in Wittenburg et al. (2011). Briefly, following the density and distribution of SNPs on the Illumina BovineSNP50 chip, 52,773 SNPs were simulated on the cattle genome of 30 Morgan length. Then, 400 generations of random mating among 100 individuals were executed in which random recombination events according to the genetic distance between SNPs and random mutation of SNP alleles were considered to reach a realistic amount of linkage disequilibrium between SNPs. Four more generations were produced; in every generation 50 sires were mated to 20 dams in order to generate multiple half‐sib families. The data were split into training (generations 401 and 402, n=2,000) and validation set (generations 403 and 404, n=2,000). Then, p=5,227 SNPs (every 10th SNP including the causative variants) were used for the evaluation. SNPs with MAF ⩽0.05 were removed, and thus 793 SNPs were additionally excluded on average. Twenty‐three SNPs were randomly preselected to be the causative variants, and additive and dominance effect were simulated for each of them. For each of the four kinds of epistatic effects, six SNP pairs were randomly drawn out of the 23 causative variants to interact. Dominance and epistasis explained about 10% and 29%, respectively, of the total genetic variance. Two different traits were achieved by adding different residual error terms to the sum of genetic effects, such that total genetic variation contributed either 30% (i.e. broad‐sense heritability $H^{2}=0.3$) or 50% ($H^{2}=0.5$) to the variation of ***y***. The simulation was repeated 100 times. The fastbayes algorithm was executed using γ=0.005, $\lambda =\sqrt{2p\gamma }$ for additive and dominance effects and $\gamma =10^{-6}$, $\lambda =\sqrt{p(p-1)\gamma }$ for epistatic effects, as suggested by Wittenburg et al. (2011).

The main criterion for evaluation was the number of truly detected SNPs with significant impact on a trait. This was measured in terms of sensitivity and specificity for each kind of effect separately as well as overall by considering the joint set of SNPs that were significant in any kind of effect. A true positive result was obtained when the significant SNP was in a window of 100 kbp around the simulated causative variant. For instance, one gene per 100 kbp is expected in the mouse genome (Laurie et al., 2007). The impact of different values for the type‐I error on sensitivity and specificity was investigated using α∈{0.01,0.05,0.10,0.20}. Furthermore, the proportion of estimated genetic variance explained by significant SNPs to the simulated genetic variance ($\sigma_{\text{sign}}^{2}$) was calculated. These characteristics determine the ability of an approach to select the loci relevant to genetic variation in a training set. Moreover, the ability to predict genetic values of future or not‐yet phenotyped animals was verified. Accuracy of genetic value prediction was assessed as the correlation between estimated and simulated genetic values in a test set.

A permutation test approach was used to further study the sensitivity and specificity of fastbayes in the absence of any effects of causative variants. The association between genotypes and phenotypes was removed in the first simulated dataset by shuffling the SNP genotypes: the rows of the matrix *X* were randomly assigned to the animals. This was repeated 100 times, and the resampled datasets were analyzed as described above.

### 3.2 Real data

To show the performance of fastbayes on real data, data of a heterogeneous stock of mice (Valdar et al., 2006a) retrieved from http://gscan.well.ox.ac.uk on July 12, 2011 were analyzed. The dataset comprised genotypes at p=8797 SNPs (MAF >0.05) and phenotypes of n=1521 animals. Rarely missing genotypes for these SNPs were imputed via Beagle 3.2 (Browning & Browning, 2007). The percentage of CD8^+^ cells, an immunological phenotype, was analyzed, and the vector of observations was standardised to avoid numerical problems. A set of covariates similar to Valdar et al. (2006b) was considered: gender, age, family, litter, cage density, experimenter, month, and year of experiment. A resampling scheme was used to specify a suitable hyperparameter γ. The data were equally split into training and test set, and animals were assigned at random to the sets. For a range of γ∈[0.001,0.1], genetic values (EGV) were estimated in the test set based on the effect estimates from the training set using a model with additive and dominance effects. The correlation between EGV and ***y*** served as a measure of accuracy. The γ leading to the highest accuracy was chosen. Then, to specify the hyperparameter for epistatic effects, a range of $\gamma \in \{10^{-7},\ldots ,10^{-3}\}$ was evaluated similarly. For instance, 10^−7^ corresponds to an expectation of four interactions per kind of effect. Using a smaller γ‐value would reflect the assumption of less than one interaction effect that is not envisaged. The final parameter values were employed in the genomic evaluation of the complete dataset.

## 4 SOFTWARE IMPLEMENTATION

The data preparation, analysis and summary of results were executed in R version 3.5.0 (R Core Team, 2015). The fastbayes approach was implemented in Fortran90 embedding CDFLIB routines (http://biostatistics.mdanderson.org/SoftwareDownload/); fastbayes is available online at https://github.com/wittenburg/fastbayes. The search for the HPD interval was implemented as a grid search over the interval $\left[m_{L},m_{U}\right]$ with $m_{L}=10\min\left(\hat{g}\right)$ and $m_{U}=10\max\left(\hat{g}\right)$; the step size was $(m_{U}-m_{L})/2\;000$. The dummy variables in $X_{e}$ were set up on the fly to reduce memory allocation. The fastbayes was compared to Logsdon's vbay method (Logsdon et al., 2010) being the strongest competitor in terms of computing time and accuracy of estimation; it is available as R package vbsr version 0.0.5 (Logsdon, Carty, Reiner, Dai, & Kooperberg, 2012). The vbay's approximate posterior probability of a parameter being non‐zero was used to assess significance. If this probability was >0.95, a significant effect was reported. The calculations were run on 2.1 GHz (SLES 12 64 bit) and 2.2 GHz (SLES 12 SP 3 64 bit) multiuser systems.

## 5 COMPUTING DETAILS

Computing time was clearly in favor of fastbayes; it generally needed the least time, see Table 1. The differences in time between fastbayes and vbay were small when additive and dominance effects were studied in simulated data. For estimating approximately 39 million epistatic effects and computing their significance measures, fastbayes required on average 5.4 hr when $H^{2}=0.5$. More iterations were needed until convergence when $H^{2}=0.3$; then computations took 5.9 hr on average. Due to memory restrictions in the Fortran implementation used in the R package for vbay (long vectors were not supported), the full model containing 2p(p−1) pairwise interactions could not be analyzed. To obtain a tendency whether vbay is able to reveal epistatic effects, the model dimension was further decreased. Again, every 10th out of the 5,227 SNPs was selected but without paying attention to keeping the causative variants among the selected SNPs. Considering MAF >0.05, 444 SNPs were retained on average. Additive and additive× additive interaction effects were analyzed, denoted as model M3* in Table 1. In this downsized scenario, fastbayes needed 2 min computing time but 10 min were required by vbay for estimating 98,790 effects.

**Table 1:** Average computing time of a single analysis based on the fastbayes and vbay approach

Model	fastbayes	vbay
	Simulated data (⌀ 4,434 SNPs)	
M1	4 s	14 s
M2	7 s	29 s
M3^*^	2 min	10 min
M3	5.4 hr	–
	Real data (8,797 SNPs)	
M2	10 s	26 min
M3	16.9 hr	–

M1: model with additive effects; M2: model with additive and dominance effects; M3: model with additive, dominance and all epistatic effects; M3*: model with additive and additive× additive interaction effects using only every 10th SNP.

The difference in time increased in the real data analysis (Table 1). Additive and dominance effects were computed in 10 s using fastbayes and 26 min using vbay. Furthermore, 16.9 hr were required for estimating about 154 million interaction effects. Due to memory restrictions, vbay could not be used for studying epistatic effects.

Because a multiuser computing system was employed, the memory allocation could only be approximated roughly for simulated data. Peaks in memory allocation have been observed when epistatic effects were estimated using fastbayes (⩽4 GB on average) and when main genetic effects were computed using vbay (⩽1 GB on average).

## 6 RESULTS OF GENOMIC EVALUATIONS

### 6.1 Simulated data

Considering the measure of evidence, the fastbayes approach identified about one‐third of the causative variants with additive contribution to the total genetic variation if $H^{2}=0.5$, see Table 2. If α=0.01, sensitivity for additive effects increased slightly by about 6% when the genome‐wide regression model was extended to include dominance effects but these were rarely identified; the sensitivity for dominance effects was 7%. The best outcome was observed if pairwise epistatic effects were also included in the model. Then, considering the joint set of significant SNPs over all kinds of effects, 50% of the causative variants were identified correctly. The higher the type‐I error was, the higher the sensitivity turned out; the maximum was observed if α=0.20. The specificity, however, remained very high (≥99.76%) in general. Larger α‐values led to a further but very small increase of sensitivity (results not shown), and specificity was still very high (e.g. ≥99.74% if α=0.50). This can be explained by the clear differentiation of zero and nonzero effects by the measure of evidence, see Additional File 1: effects close to zero coincidentally have a measure close to one. The genetic variance that could be explained by the significant effects was 89% at most. The accuracy of genetic value prediction achieved its maximum over all models at 82%.

**Table 2:** Average sensitivity for each kind of effect and overall specificity based on the fastbayes (measure of evidence MOE; Bayes factor BF) and vbay approach, $H^{2}=0.5$

		Sensitivity				Specificity	
	Model	*a*	*d*	*e*	Overall	overall	$\sigma_{\text{sign}}^{2}$
fastbayes	M1	0.315			0.315	1.000	0.536
(MOE ⩽0.01)	M2	0.334	0.071		0.369	1.000	0.601
	M3	0.373	0.107	0.246	0.504	0.999	0.833
fastbayes	M1	0.337	–	–	0.337	1.000	0.545
(MOE ⩽0.05)	M2	0.352	0.090	–	0.393	1.000	0.615
	M3	0.390	0.116	0.256	0.522	0.998	0.872
fastbayes	M1	0.347	–	–	0.347	1.000	0.550
(MOE ⩽0.10)	M2	0.361	0.098	–	0.406	1.000	0.622
	M3	0.394	0.121	0.256	0.525	0.998	0.884
fastbayes	M1	0.360	–	–	0.360	1.000	0.555
(MOE ⩽0.20)	M2	0.369	0.107		0.417	1.000	0.628
	M3	0.397	0.123	0.256	0.527	0.998	0.891
fastbayes	M1	0.398	–	–	0.398	0.999	0.560
(BF >3)	M2	0.407	0.149	–	0.467	0.998	0.639
	M3	0.418	0.153	0.273	0.563	0.994	0.927
vbay	M1	0.356	–	–	0.356	1.000	0.587
	M2	0.357	0.094	–	0.400	1.000	0.654

M1: model with additive (*a*) effects; M2: model with additive and dominance (*d*) effects; M3: model with additive, dominance and epistatic (*e*) effects; contribution of the variance at the significant SNPs to the total genetic variance ($\sigma_{\text{sign}}^{2}$). In total, 23 causative variants were simulated.

The overall sensitivity of fastbayes further improved by about 7−12% when the Bayes factor instead of the measure of evidence (α=0.20) was used for identifying the relevant SNPs. Both measures found the same significant effects but few additional effects, which were most often nonadditive, could be identified with the Bayes factor. The best result was achieved with the genome‐wide regression model including all kinds of genetic effects. Then, 42% of the causative variants with additive effects were detected on average. The sensitivity for dominance effects increased to 15% but sensitivity for epistatic effects remained rather constant at 27%; 56% of the causative variants were found overall, see Table 2. The overall specificity was ≥99% being slightly less than with the measure of evidence. The genetic variance explained by the SNPs with substantial effect was 93%.

With $H^{2}=0.3$, the performance of fastbayes was worse than with $H^{2}=0.5$, see Table 3. About 30 − 33% less true positive SNPs could be identified overall using the measure of evidence and about 24−25% using the Bayes factor. The genetic variance explained by the significant SNPs decreased by 4%. The complex model including all kinds of genetic effects yielded again the best outcome regarding the ability to detect the relevant loci that capture most of the genetic variation but the accuracy of genetic value prediction was slightly reduced by 2% compared to a model containing additive and dominance effects; the correlation between simulated and estimated breeding values was 72%.

**Table 3:** Average sensitivity for each kind of effect and overall specificity based on the fastbayes (measure of evidence MOE; Bayes factor BF) and vbay approach, $H^{2}=0.3$

		Sensitivity				Specificity	
	Model	*a*	*d*	*e*	Overall	overall	$\sigma_{\text{sign}}^{2}$
fastbayes	M1	0.227	–	–	0.227	1.000	0.483
(MOE ⩽0.05)	M2	0.240	0.041	–	0.264	1.000	0.539
	M3	0.269	0.058	0.128	0.366	0.998	0.840
fastbayes	M1	0.303	–	–	0.303	0.999	0.508
(BF >3)	M2	0.305	0.088	–	0.352	0.998	0.578
	M3	0.305	0.090	0.145	0.423	0.994	0.892
vbay	M1	0.253	–	–	0.253	1.000	0.564
	M2	0.253	0.047	–	0.280	1.000	0.611

M1: model with additive (*a*) effects; M2: model with additive and dominance (*d*) effects; M3: model with additive, dominance and epistatic (*e*) effects; contribution of the variance at the significant SNPs to the total genetic variance ($\sigma_{\text{sign}}^{2}$). In total, 23 causative variants were simulated.

Furthermore, the data that were generated without any impact of the causative variants were analyzed. It turned out that sensitivity was 0.17% at most and specificity was ≥99.88% over all kinds of effects and all models using the measure of evidence and, for instance, α=0.05. Using the Bayes factor, the overall sensitivity was increased but it was at most 0.87% in the model considering all kinds of effects and specificity was ≥99.42%. The correlation between simulated and estimated breeding values was 0.13%.

The ability to select the relevant loci and to predict the genetic values was similar using fastbayes (and the measure of evidence, α=0.05) and vbay based on the model with additive and dominance effects, see Tables 2–4. Vbay, however, could explain up to 6–13% more of the total genetic variance considering the significant effects because intermediate effects were estimated negligibly better than using fastbayes (see also Additional File 1). Both methods could not detect the small effects correctly.

**Table 4:** Average sensitivity for each kind of effect and overall specificity based on the fastbayes (measure of evidence MOE; Bayes factor BF) and vbay approach in the absence of genetic effects

		Sensitivity				Specificity
	Model	*a*	*d*	*e*	Overall	overall
fastbayes	M1	0	–	–	0	1.000
(MOE ⩽0.05)	M2	0	0	–	0	1.000
	M3	0	0	0.002	0.002	0.999
fastbayes	M1	0.001	–	–	0.001	1.000
(BF >3)	M2	0.001	0.001	–	0.002	0.998
	M3	0.001	0.002	0.006	0.009	0.994
vbay	M1	0	–	–	0	1.000
	M2	0	0	–	0	1.000

M1: model with additive (*a*) effects; M2: model with additive and dominance (*d*) effects; M3: model with additive, dominance and epistatic (*e*) effects. In total, 23 causative variants were simulated.

### 6.2 Real data

**Figure 2:**
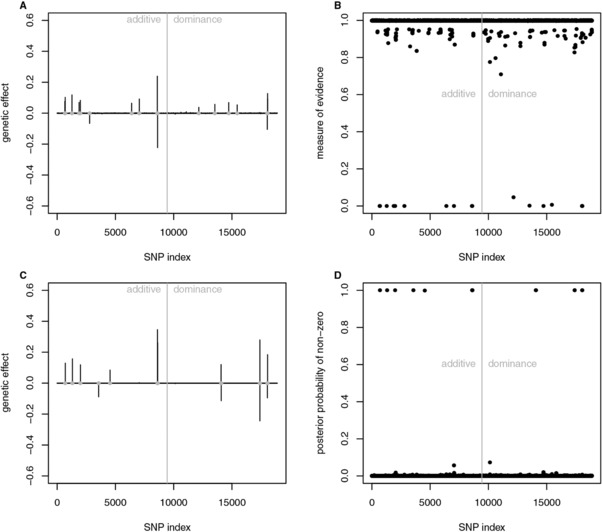
Results of analyzing the mouse data set (p=8,797 SNPs, n=1,521 individuals): estimated additive and dominance effects of SNPs using the fastbayes (A) and vbay (C) approach; gray dots indicate significant loci. Measure of evidence related to fastbayes (B) and posterior probability of nonzero effects related to vbay (D) reflect the significance of effects. SNP index equals SNP number for additive effects and SNP number plus *p* for dominance effects

The SNPs with significant effect were located near protein coding genes or in regions of quantitative trait loci (QTL) with known association to traits of the immune system (http://www.informatics.jax.org). Though results obtained by fastbayes (α=0.05) and vbay have been rather similar based on simulated data, the real data analysis revealed differences regarding the dominance effects, see Figure 2 and Additional File 2. With vbay, two significant dominance SNPs were detected on chromosome 17 near the MHC region which has great impact on immunological phenotypes (Valdar et al., 2006a). The largest dominance effects (contributing 26% to the total genetic variance) were found on chromosome 15; this region was not identified by fastbayes. Fastbayes estimated a group of three SNPs near the MHC region with similar, moderate dominance effects and smaller effects on the other chromosomes. The SNPs near the MHC region also had the largest additive impact (50%) on the total genetic variance. Other SNPs with moderate additive effects were found on chromosome one and two and were in the neighborhood of known QTL (*Sle1c* and *Prdt1*, respectively). The significance measures of both methods clearly differentiate between significant and nonsignificant effects (see Figure 2B and D). In total, vbay identified eight loci with additive and six loci with dominance effect. These loci explained all of the estimated genetic variance. Fastbayes detected 10 loci with additive and seven loci with dominance effect using an optimal γ=0.046, and 99% of the estimated genetic variance was explained by the significant effects. This outcome did not change using the complex model including epistasis – no significant epistatic effects were found. The optimal hyperparameter for epistatic effects was $\gamma =10^{-7}$.

**Figure 3:**
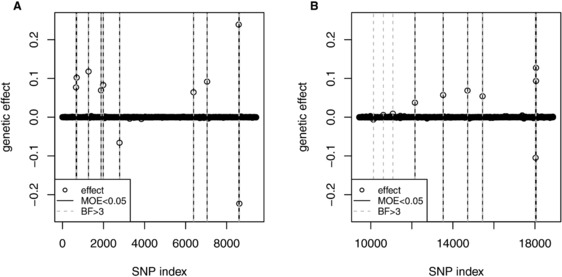
Results of analyzing the mouse dataset (p=8,797 SNPs, n=1,521 individuals): significance of additive and dominance effects of SNPs was inferred using fastbayes and (A) measure of evidence (MOE) ⩽0.05 or (B) Bayes factor (BF) >3. SNP index equals SNP number for additive effects and SNP number plus *p* for dominance effects

Using the Bayes factor for inferring significance again showed that the same significant additive effects were identified as with using the measure of evidence but additional nonadditive effects could be detected. For instance, in Figure 3B, three more dominance effects were found on chromosomes 1–3. However, each of them explained less than 0.02 % of the genetic variance and might be negligible.

## 7 DISCUSSION

This study revisited the approximate Bayesian approach “fastbayes” that was designed for genomic evaluations. The suitability of this approach in terms of accuracy of estimating genetic values and genetic variation has been elucidated earlier, and it has been compared to an MCMC‐based method in Wittenburg et al. (2011). In this study, it was shown that the extension of fastbayes to include a marginal significance test for SNP effects, which is theoretically founded, enables the detection of loci relevant to genetic variation. Though the specificity of the corresponding measure of evidence was hardly affected by changes in the type‐I error level, sensitivity was better at higher levels, for instance α=0.20. Particularly regarding the nonadditive effects, sensitivity was even larger when the Bayes factor was used but then specificity was slightly decreased. However, its benefit was leveled in real data investigations. Sensitivity and specificity were also similar to a variational Bayesian method “vbay” (Logsdon et al., 2010). Fastbayes required less computing time than vbay.

For the identification of loci with significant additive or nonadditive genetic impact on a trait, MCMC‐based stochastic variable selection methods are useful (e.g. Bennewitz, Edel, Fries, Meuwissen, & Wellmann, 2017; Yi et al., 2005). Depending on the number of iterations and on the model dimension, such methods are exact but may need exhausting computing time. Pairwise interaction effects lead to millions of model parameters to be estimated. To avoid this, flexible model reduction techniques exist. For instance, the reversible‐jump technique can be used to avoid an oversaturated model (Balestre & de Souza, 2016). As mixing can be poor in reversible‐jump MCMC algorithms (Hastie, 2005), other possibilities are sought for identifying the relevant loci. Using a single‐marker regression model, which may be combined with a feature‐ranking step for main and epistatic effects, is rather common in genome‐wide association studies. A parallel (Schüpbach, Xenarios, Bergmann, & Kapur, 2010) and GPU‐based (Kam‐Thong et al., 2012; Ueki & Tamiya, 2012) implementation allow quickly scanning the genome for significant effects in ultra‐high dimensions. Fastbayes differs from such a GWAS approach: while a single parameter (i.e. the effect of a single SNP or SNP pair) is considered successively, the vector of observations is marginalised (i.e. corrected for all other temporarily estimated effects) in an iterative manner. Thus, at each stage of iteration, the full genome is considered which should lead to a more precise localization of relevant loci. Considering additional kinds of genetic effects in a whole‐genome regression model improves the power to map loci which are relevant to genetic variation in general. This was observed for the approximate Bayesian approaches used in this study, for MCMC‐based methods (e.g. Bennewitz et al., 2017) and for other SNP‐selection approaches (e.g. Sabourin, Nobel, & Valdar, 2015). However, sample size matters when nonadditive effects are studied (e.g. Van Steen, 2011).

As an option, a GWAS approach based on a single‐marker regression model that considers the empirical correlation among SNPs (Zuber et al., 2012) was applied to the simulated data; it is available as R package care version 1.1.9. The top‐ranked SNPs corresponding to a false discovery rate of 5 % were selected as significant. The rate of true positive detections was lower compared to fastbayes and vbay: sensitivity for additive effects was 26 %, for dominance 9 % and overall 32 % when $H^{2}=0.5$. The computation required 9 min on average.

The specification of hyperparameters may have a great impact on the outcome of Bayesian approaches (e.g. Tempelman, 2015). For the analysis of simulated data, the parameter γ specifying the proportion of mixing a zero and nonzero distribution of SNP effect was fixed near its true value and $\lambda =\sqrt{2p\gamma }$ like in Wittenburg et al. (2011). For the real data analysis, a simple attempt was made to specify γ depending on the data. A more extensive approach is to execute cross validation like in Melzer, Wittenburg, and Repsilber (2013). As an option toward a fully Bayesian approach, this parameter could be modeled additionally. For this purpose and similar to Scott & Berger (2006), it is assumed that γ is small. Then a suitable choice of the prior density, which allows a reasonable amount of variation, is $p\left(\gamma \right)=\left(a+1\right){\left(1-\gamma \right)}^{a}$ with some prior information *a*. The posterior expectation has been worked out in Additional File 3 and has been incorporated in the fastbayes algorithm. As the distribution of γ varies only little, the estimates of genetic effects and their measure of significance were not altered seriously in case of simulated data. As an example for $H^{2}=0.5$ and based on the complex model including all kinds of genetic effects, on average one to two loci were additionally detected but the number of truly detected loci was almost unchanged. The sensitivity over all kinds of effects was 52 %. The significant loci explained about 90 % of the total genetic variance. The accuracy of genetic value prediction was 80 %.

Not surprisingly, the real data analysis of a heterogeneous stock of mice revealed results similar to Wittenburg et al. (2011). However, now the measure of evidence introduced in Section 2 rose clearly, proving the significance of the largest effects. Unlike Wittenburg et al. (2011), γ was specified based on a resampling approach. As a consequence, γ was increased for main effects and decreased for epistatic effects. Then more medium additive and dominance effects but no epistatic effects were detected in this study. The large dominance× additive epistatic effect that was reported earlier probably split into an additional additive and dominance effect.

Fastbayes is developed for, but not limited to, applications in animal breeding. It is straightforward to be applicable in plant breeding, where genome‐based selection of phenotypes is an on‐going issue for efficient plant production (e.g. Desta & Ortiz, 2014). This approach does not account for any population structure that might appear in a livestock population. But population stratification may cause biased allele frequency estimates used to set up *X*. As human genetic datasets typically consist of many unrelated individuals, this approach is perfectly suited for GWAS in human genetics. There, the elucidation of the genetic architecture of common diseases (e.g. Ripke et al., 2013) or other traits (e.g. Pickrell et al., 2016) is targeted, and the genetic merit is not focused. Furthermore, genetic data from human sibling studies can also be processed. Such data allow the precise estimation of the genetic impact of complex traits (e.g. Sariaslan et al., 2016). They are valuable to estimate the contribution of dominance to the phenotypic variation, too.

As discussed in Van Steen (2011), prior knowledge, which may be derived from biological information, would enhance the interpretation of results of genomic evaluations. Such information can be retrieved from data bases, networks or pathway analyses and can be included in a statistical model. As an option, this knowledge may be translated into weights $w_{j}\in \left[0,\infty \right)$ for each locus or kind of effect j=1,2,…, where zero means exclusion and one the neutral weight. Similar to a weighted regression analysis, a vector of covariates $x=X_{j}\sqrt{w_{j}}$ is considered in the one‐locus model (2). This strategy of weighting has already been implemented in the fastbayes algorithm but it still needs to be validated.

## 8 CONCLUSIONS

The extension of the approximate Bayesian approach “fastbayes” to include a marginal measure of significance allows now a quick scan of the genome to identify the SNPs that are relevant to genetic variation. The clear benefit of fastbayes is its dual‐purpose use: it estimates the effect sizes for all kinds of genetic effects (additive and nonadditive) and determines the significant SNPs or SNP pairs. It requires much less computing time in higher, realistic dimensions than other approaches for whole‐genome regression analyses. Due to its speed, it will also be valuable for the analysis of whole‐genome sequence data.

## Supporting information

Supplementary Material

Supplementary Material

Supplementary Material

Supplementary Material
