# Supplementary material for: An approximate Bayesian significance test for genomic evaluations
Source: Biom J. 2018 Aug 12;60(6):1096–109. doi: 10.1002/bimj.201700219 (PMC6282823; doi:10.1002/bimj.201700219)
Supplement: Supplementary file 1 — Supplementary Material [file BIMJ-60-1096-s001.pdf]

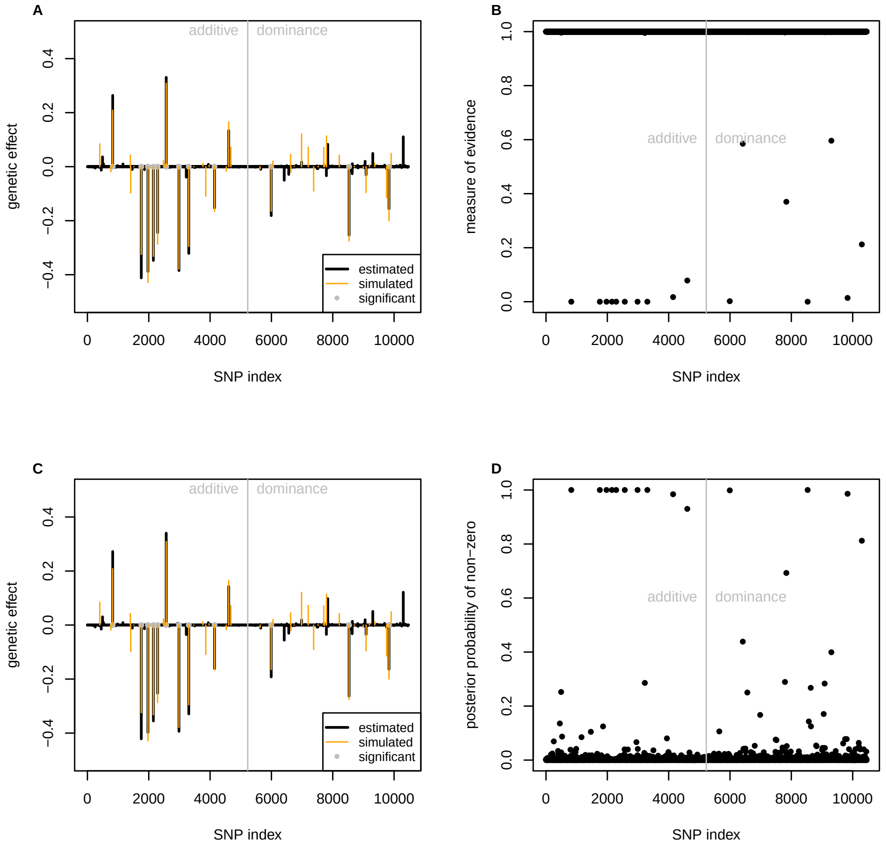

Figure A1: Results of analysing a single simulated data set ($p = 4\,448$ SNPs, $n = 2\,000$ individuals). Estimated additive and dominance effects of SNPs using the fastbayes (A) and vbay (C) approach. Measure of evidence related to fastbayes (B) and posterior probability of non-zero effects related to vbay (D) reflect the significance of effects. SNP index equals SNP number for additive effects and SNP number plus p for dominance effects. In total, 23 causative variants were simulated and $H^2 = 0.5$.
