# Supplementary material for: An approximate Bayesian significance test for genomic evaluations
Source: Biom J. 2018 Aug 12;60(6):1096–109. doi: 10.1002/bimj.201700219 (PMC6282823; doi:10.1002/bimj.201700219)
Supplement: Supplementary file 2 — Supplementary Material [file BIMJ-60-1096-s002.pdf]

fastbayes: additive			
name	chr	cM	var.add
rs3657320	1	89.649941	0.027522
rs13476251	1	95.525741	0.048222
rs4223428	2	66.418533	0.064450
rs13477269	3	43.470183	0.022356
rs13477354	3	55.664668	0.031980
rs6230717	4	92.247953	0.020309
rs13481363	12	11.354773	0.019202
rs6245977	13	45.125676	0.038744
CEL-17_31069801	17	15.630574	0.266384
mCV22965443	17	16.847716	0.230393
fastbayes: dominance			
name	chr	cM	var.dom
rs4138996	4	78.330039	0.006547
rs3675028	7	33.238675	0.015160
rs3089531	9	61.853809	0.021900
rs3668680	11	30.046139	0.013376
CEL-17_31069801	17	15.630574	0.051199
mCV22965443	17	16.847716	0.074637
mhcTNFa7	17	16.901652	0.040449
vbay: additive			
name	chr	cM	var.add
rs3723788	1	96.523035	0.031345
rs13476764	2	68.049852	0.046258
rs6156541	3	56.751170	0.026414
mCV23348277	6	35.560927	0.014601
CEL-8_36058876	8	21.660969	0.013370
rs3023450	17	16.197795	0.223679
rs8258655	17	16.201180	0.127189
rs8237882	17	18.051537	0.127855
vbay: dominance			
name	chr	cM	var.dom
rs6398181	8	29.450794	0.027016
mCV22586111	8	30.505905	0.024202
rs13482630	15	32.387427	0.146003
rs13482631	15	32.400679	0.111489
rs3724223	17	16.104732	0.016999
mCV22965443	17	16.847716	0.063487

Table A1: List of significant SNPs and their location (mouse map build 3.7) detected by the fastbayes ($\alpha = 0.05$) and vbay approach. Results are based on the real data analysis of the heterogeneous stock of mice. The contribution of variation of the additive and dominance effects to the total genetic variance is given.
