# Supplementary material for: An approximate Bayesian significance test for genomic evaluations
Source: Biom J. 2018 Aug 12;60(6):1096–109. doi: 10.1002/bimj.201700219 (PMC6282823; doi:10.1002/bimj.201700219)
Supplement: Supplementary file 3 — Supplementary Material [file BIMJ-60-1096-s003.pdf]

Estimation of the proportion of non-zero effects

Similar to Scott and Berger (2006), it is assumed that γ is small. Then a suitable choice of the prior density, which allows a reasonable amount of variation, is

$$p(\gamma) \quad = \quad (a+1)(1-\gamma)^a$$

with some hyperparameter $a > 0$. For estimating $\widehat{\gamma} = E(\gamma|\boldsymbol{y})$ the marginal distribution $p(\gamma|\boldsymbol{y})$ is required,

$$\begin{aligned} p(\gamma|\boldsymbol{y}) &= \frac{1}{p(\boldsymbol{y})} \int\limits_{\mathbb{R}} p(\boldsymbol{y}|g)p(g|\gamma)p(\gamma)dg \\ &= \frac{1}{p(\boldsymbol{y})} \int \left[f_1(g)\mathbb{1}_{\{g<0\}} + f_2(g)\delta_0(g) + f_3(g)\mathbb{1}_{\{g>0\}}\right](a+1)(1-\gamma)^a dg\,, \end{aligned}$$

with functions f_1, f_2, f_3 as in section "Methods" of the main paper. The conditional expectation is then

$$\begin{aligned} \widehat{\gamma} \;=\; E(\gamma|\boldsymbol{y}) &= \frac{1}{p(\boldsymbol{y})} \int\limits_0^1 \gamma\, p(\gamma|\boldsymbol{y})d\gamma \\ &= \frac{1}{p(\boldsymbol{y})} \frac{2}{(a+3)(a+2)} \left[\frac{1}{2}\lambda \exp\left(\frac{1}{2}\lambda^2\sigma^2\right)(T_1+T_2) + \frac{a+1}{2}\phi(0;Y,\sigma^2)\right] \end{aligned}$$

with T_1, T_2 as above and

$$\begin{aligned} p(\boldsymbol{y}) &= \int\limits_0^1 \int\limits_{\mathbb{R}} p(\boldsymbol{y}|g)p(g|\gamma)p(\gamma)dg\,d\gamma \\ &= \int\limits_0^1 \left\{\frac{1}{2}\gamma\lambda \exp\left(\frac{1}{2}\lambda^2\sigma^2 + \lambda Y\right)\Phi(0;Y^+,\sigma^2)\right. \\ &\quad +(1-\gamma)\phi(0;Y,\sigma^2) \\ &\quad \left.+\frac{1}{2}\gamma\lambda \exp\left(\frac{1}{2}\lambda^2\sigma^2 - \lambda Y\right)\left[1-\Phi(0;Y^-,\sigma^2)\right]\right\}(a+1)(1-\gamma)^a d\gamma \\ &= \frac{1}{a+2}\left[\frac{1}{2}\lambda \exp\left(\frac{1}{2}\lambda^2\sigma^2\right)(T_1+T_2) + (a+1)\phi(0;Y,\sigma^2)\right]. \end{aligned}$$

The hyperparameter a may be chosen such that

$$\begin{aligned} E(\gamma) &= \int\limits_0^1 p(\gamma)d\gamma \\ &= \frac{1}{a+2} \stackrel{!}{=} \gamma_0 \\ \Rightarrow\; a &= \frac{1}{\gamma_0} - 2 \end{aligned}$$

with some prior guess γ_0.

Eventually, γ is estimated iteratively for each locus and for each kind of effect within the Gauss-Seidel-like algorithm.

References

Scott, J.G. and Berger, J.O. (2006) An exploration of aspects of Bayesian multiple testing. *Journal of Statistical Planning and Inference*, **136**, 2144–2162.
